# Is the Diagnosis of Generalized Stage IV (Severe) Periodontitis Compatible with the Survival of Extended Stabilizing Prosthetic Restorations? A Medium-Term Retrospective Study

**DOI:** 10.3390/diagnostics12123053

**Published:** 2022-12-05

**Authors:** Viorelia Rădulescu, Marius Boariu, Darian Rusu, Camelia Boldeanu, Ruxandra Christodorescu, Alexandra Roman, Petra Surlin, Andreea Cristiana Didilescu, Octavia Vela, Giorgios Kardaras, Ioana Veja, Ioana Martu, Stefan-Ioan Stratul

**Affiliations:** 1 Department of Periodontology, Faculty of Dental Medicine, Anton Sculean Research Center for Periodontal and Peri-Implant Diseases, “Victor Babes” University of Medicine and Pharmacy, 300041 Timisoara, Romania; 2 Department of Endodontics, Faculty of Dental Medicine, TADERP Research Center, “Victor Babes” University of Medicine and Pharmacy, 300041 Timisoara, Romania; 3 Department V Internal Medicine, Faculty of Medicine, “Victor Babes” University of Medicine and Pharmacy, 300041 Timisoara, Romania; 4 Department of Periodontology, Faculty of Dental Medicine, Applicative Periodontal Regeneration Research Unit, Iuliu Hatieganu University of Medicine and Pharmacy, 400012 Cluj Napoca, Romania; 5 Department of Periodontology, Faculty of Dental Medicine, University of Medicine and Pharmacy, 200349 Craiova, Romania; 6 Department of Embryology, Faculty of Dentistry, Carol Davila University of Medicine and Pharmacy, 8 Eroii Sanitari Boulevard, 050474 Bucharest, Romania; 7 Department of Dental Technology, Faculty of Dental Medicine, Grigore T. Popa University of Medicine and Pharmacy, 700115 Iasi, Romania

**Keywords:** periodontitis, dental prostheses, survival analysis, dental abutment

## Abstract

The aim of the study was to identify the most relevant patient-related factors directly involved (alone or in combinations) in the long-term survival and functionality of the abutment teeth of extensive stabilizing bridges and removable prosthesis, in patients treated for Stage IV periodontitis, adhering to SPT over a period of at least 5 years. Seventy-six patients treated between 2000–2022, rehabilitated with FDPs and RDPs, adhering to SPT for at least 5 years were included. Patient-related factors influencing retention of RDPs and FDP, survival rates in regular (RCs) and irregular compliers (ICs), and incidence of biological and technical complications were assessed. During a follow-up of 69 months, from 57 patients with FDPs and 19 patients with RDPs, 39 (51.32%) were ICs, while 37 (48.68%) were RCs. An overall statistically significant association (*p* = 0.04) was identified between biological complications and the type of prostheses. The RDP patients had more complications than FDP patients. In 5.26% of the RDP patients, root caries were identified, and 10.53% were diagnosed with a periapical (endodontic) lesion, while 3.51% of the FDPS patients presented root caries. In five (6.57%) cases, abutment loss resulted in the loss of the prosthesis. Statistically significant correlations were observed between systemic diseases and tooth loss, and between type of tooth lost and the reason for tooth loss, irrespective of the type of prosthesis. A total of 66.67% of the lost incisors, 85.71% of the lost premolars, and 88.89% of the lost molars occurred due to periodontal causes. Furthermore, 93% of the FDPs and RDPs were still in place and in function.

## 1. Introduction

The main objective of periodontal therapy is the long-term retention of as many teeth as possible in a healthy, functional, aesthetically acceptable, and painless state [1,2]. The true sequela of periodontal disease is tooth loss and, thus, the outcome of periodontal therapy and maintenance over years should be assessed by evaluating tooth loss [3,4,5].

As only a reduced group of patients experience the majority of tooth losses in a population of regular maintained periodontal subjects, it is important to differentiate these patients from those where tooth retention is likely to be unfeasible, in order to prevent premature tooth removal [6]. Tonetti et al. (2000) reported that in 273 patients, 8.8% experienced tooth loss during SPT over 5.6 years, while 54% had teeth extracted during active periodontal therapy (APT) [7]. This is similar to another study, where 50% of the teeth extracted were lost during APT and 49.8% during SPT [5].

Even teeth initially classified as questionable or hopeless [2,8] can be retained in the majority of patients [9]. Extractions of putative hopeless teeth may create the need for further prosthetic treatment [10]. Some of these ‘‘hopeless’’ teeth may have had a better prognosis than those teeth that had to be used as abutments after performing the extraction [1]. Teeth affected by severe periodontal bone loss extending to the apical portion of the root, and/or history of multiple tooth loss, tooth hypermobility due to secondary occlusal trauma and the sequelae of tooth loss, and posterior bite collapse all characterize Stage IV periodontitis [11]. Therefore, case management requires stabilization of such teeth and restoration of masticatory function.

Moreover, with reasonable limits, the amount of remaining periodontal support cannot be considered a decisive factor to predict the capability of a tooth to serve as an abutment for fixed dental prostheses (FDPs) [12]. Using a splint system or stabilizing fixed dental prostheses for joining teeth with reduced periodontal support, to avoid occlusal overload and secondary trauma, is an important method used to decrease mobility [13]. Cross-arch FDPs on abutment teeth with reduced but favorably distributed support can withstand normal occlusal forces, and do not impair closing and chewing patterns [14,15,16,17,18]. A higher rate of tooth loss was observed for patients with stabilizing bridges compared with maintenance patients not treated with bridgework [19]. It has been reported that removable dental prosthesis (RDPs) are associated with a smaller rate of tooth survival than FDPs [20]. The design of RPDs and the number of supporting teeth has been shown to influence abutment tooth loss [21,22]. A recent study reported that cumulative survival rates of tooth abutments ranged from 68.9% to 95.1% of 5 to 10 years in periodontal patients rehabilitated with RDPs [23]. A systematic review revisiting the classic prosthetic planning postulate known as “Ante’s Law” [24] found that an adequate control of periodontitis, a strict adherence to SPT and a rigid splinting of mobile abutment teeth demonstrated an estimated 10-year survival rate of almost 93% of FDPs in subjects with treated generalized severe periodontitis, and reduced periodontal support [25]. 

Although earlier studies showed that periodontal tissues around teeth serving as abutments for fixed bridgework do not react to treatment in a different way than the supporting tissues around “non-abutment” teeth [26], it is known that prosthodontic treatment increases the risk for tooth loss [1,20]. Periodontally compromised, but periodontally treated patients with prosthodontic treatment are associated with a higher risk for further tooth loss than patients without prosthodontic treatment, if biomechanical factors, such as splinting, are not considered [20]. For cross-arch FDPs in such patients, the abutment tooth loss is relevant in assessing the longevity of the FDPs [12,20], while the number of supporting teeth seems also to play a role [22]. 

Regular participation in SPT of patients with periodontally treated teeth initially considered as hopeless is of paramount significance [1], although it has been reported that teeth with severely altered periodontal support are at high risk of loss, even with SPT performed by periodontists [27]. The literature identified factors characterizing periodontal at-risk patients, namely smoking [19,28,29,30,31,32], irregular SPT [10,32], and diabetes mellitus [27]. Furthermore, tooth-related factors have been shown to influence tooth loss, namely periodontal bone loss [27,28,30], tooth mobility [27,28], furcation involvement [28,30], tooth type [33], and tooth vitality [27]. Other parameters may be influencing factors, such as type of bone loss (vertical/horizontal), use as abutment tooth, and root grooves [1]. Furthermore, a number of complications, such as caries, endodontic problems, tooth or root fractures, tooth loss, and metal or porcelain fractures have been described for large bridges [34,35,36,37,38]. All aforementioned studies neither precisely describe the severity of the periodontal disease, nor correlate the prosthodontic approach with the survival rate of the previously periodontally treated abutment teeth. The long-term results and the impact of treatment in patients with Stage IV periodontitis were only separately covered in the very recently published EFP Clinical Practice Guideline [39], so the literature regarding the stability of prosthetic rehabilitations in patients specifically diagnosed with and treated for Stage IV periodontitis is scarce.

The aim of the present study was to identify the most relevant patient-related factors directly involved (alone or in combinations) in the long-term survival and functionality of the abutment teeth for extensive stabilizing bridges and removable prosthetic constructions in patients treated for Stage IV periodontitis, adhering to SPT over a period of at least 5 years. Further outcomes, such as survival rates and incidence of biological and technical complications of FDPs and RDPs on abutment teeth with severely reduced, but healthy periodontal tissue support, were also assessed.

## 2. Materials and Methods

### 2.1. Study Population

The study was approved by the Ethical Committee of Scientific Research of the Victor Babes University of Medicine and Pharmacy, Timisoara (approval no. 27/14.01.2021). The study protocol respected the ethical principles of research on human subjects, and data confidentiality was guaranteed. 

This retrospective survey was built on a database of patients treated for generalized Stage IV periodontitis between 2000–2022. The subjects underwent active periodontal therapy (APT) and SPT in a private practice in Timisoara, Romania. Here, APT and SPT were performed by four periodontists (SIS, OV, DR, and GK). The study population consisted of 75 patients, a sample size (68–100 patients) adopted in numerous previous studies [19,20,30,31,40]. The APT (from initial presentation to last active treatment appointment) followed a conservative regimen, according to the treatment protocols of the Department of Periodontology of the Victor Babes University of Medicine and Pharmacy, Timisoara. The number of existing teeth was determined at two time points, as follows: the first SPT appointment (T0), and the latest clinical session of the SPT (T1). The clinical records were used as source of data extraction. Clinical diagnosis was retrospectively re-formulated according to the New Classification Scheme for Periodontal and Peri-implant Diseases and Conditions (2018) [11], based on the data obtained from the initial periodontal evaluation and from that time radiographs (APT), and it was performed by the same periodontist (VR).

#### 2.1.1. Inclusion Criteria

Diagnosis of Stage IV (interdental clinical attachment loss, CAL, ≥ 5 mm, radiographic bone loss extending to the middle third of the root and beyond, ≥5 teeth lost due to periodontitis, ≥30% of teeth involved, and need for complex rehabilitation), was carried out as follows:

1. Patients that underwent APT and attended SPT in the same private practice in Timisoara, Romania;
2. Intraoral or panoramic radiograph available at T0 and T1;
3. Completion of APT at least 5 years prior to last examination. If the prosthodontic reconstruction was lost after fewer than 5 years, the respective period of function was included in the analysis;
4. Extensive FDPs (bridge with at least six units, i.e., abutments and pontics, and supported exclusively by teeth) and/or RDPs with at least four abutment teeth.

#### 2.1.2. Exclusion Criteria

1. Exclusively- or combined implant-supported FDPs and RDPs;
2. Re-examination period ≤ 5 years, or discontinuation of therapy by the patient.

**Figure 1:**
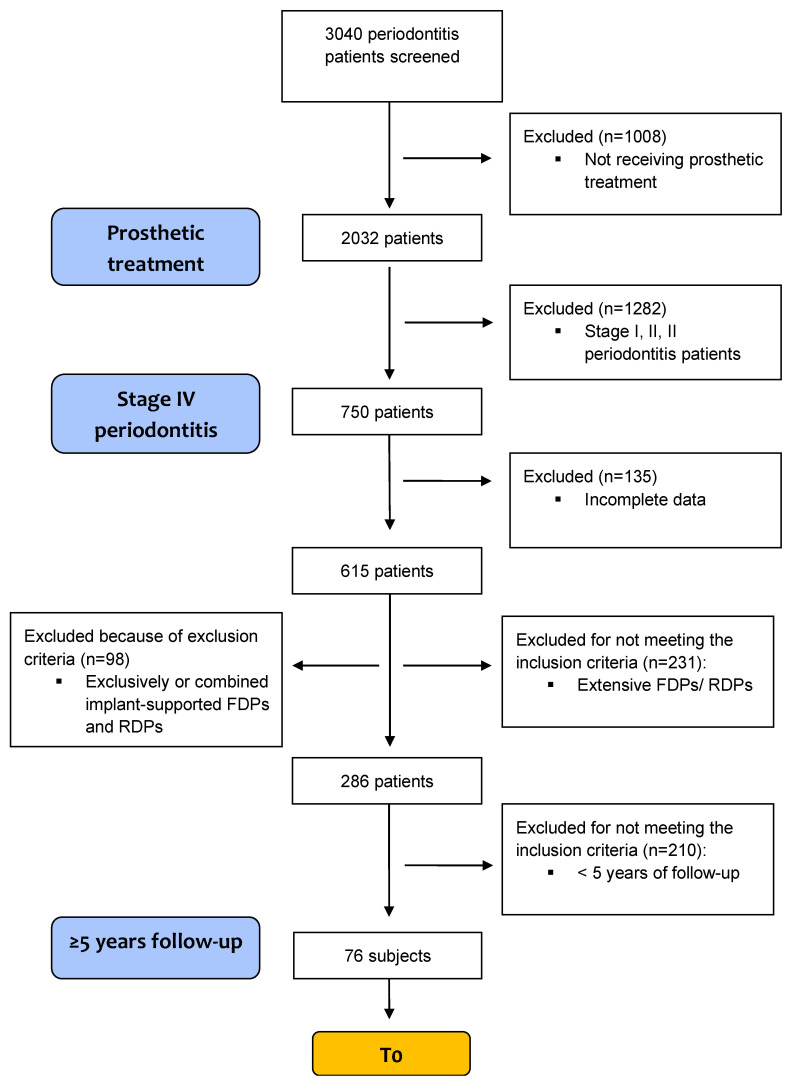
Study flow diagram based on inclusion and exclusion criteria.

All patients who fulfilled the inclusion criteria were selected consecutively for the study, until 75 qualifying patients had been included. A study flow diagram based on the inclusion and exclusion criteria is provided in Figure 1.

### 2.2. Periodontal Treatment

All patients received a similar periodontal treatment. The APT consisted of deep scaling and root planing in one or two visits within 24 h, according to the full-mouth disinfection principles [41]. Interim splinting of mobile teeth was performed in some cases during Step 1 of the periodontal treatment [42]. At the reassessment 3 months after the initial evaluation, if required, periodontal surgery was carried out for patients presenting sites with bleeding on probing or persistent deep pocketing. Periodontal conventional surgery and regenerative approaches were used where necessary [43]. 

After the completion of periodontal therapy for the treatment of Stage I–III periodontitis, definitive prosthetic treatment was performed during step R (rehabilitation), as described by the recent EFP S3 level clinical practice guideline (CPG) for the treatment of stage IV periodontitis. In the population of this study, cases of type 3 and 4 Stage IV periodontitis were identified [39].

During each SPT session, the patient was re-instructed and re-motivated to obtain an effective individual plaque control. Once or twice a year, the dental status was assessed using the periodontal chart. Re-treatment, defined as treatment over and above the prescribed maintenance, was provided when judged necessary [19,44,45].

During periodontal re-evaluations, clinical parameters were measured and recorded. Full-mouth plaque scores (FMPS) were recorded at six sites per tooth. Periodontal pocket depths (PPDs) were measured at six sites per tooth using PCP-UNC15 probes (Hu-Friedy, Chicago, IL, USA). Bleeding on probing (BOP) was assessed dichotomously in six sites per tooth. Recession (REC) were recorded to the nearest millimeter at six sites per tooth. Clinical attachment loss was calculated as the sum of PPD and REC. Mobility was recorded in degrees, according to Miller’s classification system (1985) [46]. Furcation involvement (FI) was assessed according to the classification of Hamp et al. (1975) [47] by using a Nabers probe #2N hdl #7, markings 3-6-9-12 mm (Hu-Friedy, Chicago, IL, USA).

The assignment of SPT intervals was performed according to the periodontal risk assessment tool (48). Patients exhibiting ineffective plaque control (FMPS > 35%) or showing a rapid rate of progression were seen more frequently, when deemed necessary. 

### 2.3. Patient’s Charts Evaluation

The following patient-related characteristics were extracted from patient’s charts: gender, age, compliance (appointments per year), self-reported smoking status (smoker/non-smoker/former smoker), systemic condition (cardiovascular, diabetes, autoimmune), tooth loss during treatment (yes/no). Patients who had quit smoking at least 5 years ago were considered as former smokers. The rest of the patients were classified as current smokers [48]. For statistical analyses, only smoking status at T0 was used, resulting in the fact that possible changes of smoking status during T0 or T1 were ignored. Only a few changes occurred in this respect and were recorded in the patients’ chart, but it was decided not to include them in the analysis.

The following tooth-related parameters were assessed from the patients‘ charts: type of jaw, tooth type, and furcation involvement tooth mobility. Reasons of tooth loss were categorized as follows: periodontal disease progression (increase in CAL up to the moment when it was not possible to maintain tooth in function anymore), non-treatable endodontic pathological processes, fractures, endo-periodontal lesions, and others [49]. 

Patients were classified into compliers and non-compliers, as described by Costa et al. [50,51], as follows: regular compliers (RCs) were considered to be those showing 100% cooperation with the recall visits interval; irregular compliers (ICs) were considered those that missed scheduled regular visits but continued, irregularly, with their SPT appointments.

### 2.4. Evaluation of Radiographs

A full dental status was recorded once per year. Radiographic evaluation was performed at T0 and T1 using orthopantomograms. All radiographs were viewed on large computer screens (24’). In order to reformulate the diagnosis according to the classification of 2018, bone loss (BL) was determined as a percentage of the original bone level at the most affected tooth, starting 1 mm below the restoration margin, irrespective of the length of the root and its length on the radiograph and irrespective of the direction of the X-ray beam, and it was assessed digitally to the nearest 10%. A bone loss mean value was calculated using the mesial and distal measurements. All radiographic assessments were performed by the same examiner (BM). In 10 patients with actual periodontitis Stage IV, not included in the study, radiographic assessments were repeated after 14 days to assess the reproducibility (intra-examiner calibration). The intra-examiner agreement (±1 mm > 90%) was 94%. 

Recorded data also included radiolucency with the absence of a lamina dura around the apex, suggesting a chronic periapical lesion, radiolucency on the crown or on the coronal or middle third of the root aspect, suggesting dental caries, radiopacities consistent with a root canal filling, and radiopacities consistent with dental restorations.

### 2.5. Prosthetic/Rehabilitation Considerations and Follow-Up

During Step 3 of periodontal treatment, the decision to recommend stabilizing bridges was taken in the presence of the following factors: masticatory disfunction, secondary occlusal trauma, bite collapse, drifting, and flaring of the remaining teeth. The design of the prostheses was based on the numbers of the remaining teeth, on their periodontal status, and on their prosthetic value. All bridges were designed with rigid components, no stress-breakers, and were cemented. Small preparation angles were used for abutment preparation in order to create a secure retention and to reduce the incidence of the loss of retention known as a major problem of crowns fitted on periodontally compromised teeth [36]. FDPs with cantilevers were completely avoided because the danger of abutment teeth fracture in this type of constructions. To avoid biological and technical complications, restorative procedures were conducted according to the suggestions summarized by Lindhe and Nyman (2003) [52]. If possible, the preparation was conservative, and margins were located supragingivally [53]. 

The bridge(s) observation in months, the number of teeth used as bridge abutments at T0, and the number of teeth lost between the T0 and T1 were recorded. The following technical problems with the bridge frameworks during the observation period were identified: loss of retention, fracture of metal framework, fracture of the ceramic layer, and mobility of the prostheses (due to the individual mobility of abutment teeth) [25]. Biological complications affecting the teeth, including changes in PPDs, CAL, FMPS, FMBS, radiographic alveolar bone height, pulpal conditions, and incidence of root caries, were recorded. 

### 2.6. Statistical Analysis

This retrospective study was performed on a population of 76 patients, and the patient was regarded as the statistical unit. The structure of the patient sample is described in Table 1. For clinical parameters PPD and CAL, a mean value per patient was determined, and a mean value per group was used when making comparisons. The survival period of abutment teeth was counted from the entry-point (T0, baseline) defined as the moment when the FDP/RDP was cemented in place, until the end-point (T1), defined as either the date of the last visit to the dentist or the date of abutment tooth loss (defined as extraction of the tooth). The follow up period is referred as the time when at least 120 months (5 years) had passed since the date of RPD/ FDP provision. Data distributions were expressed as means, standard deviations (SD), medians, and percentages. For continuous data, comparisons were done using Student’s *t*-test and one-way ANOVA test. For categorical measures, Pearson chi-squared tests were used. The Fisher exact test was used when the expected frequency of any cell in the table was <5. For the survival analysis, the Kaplan–Meier method was used for plotting the survival curves, and the log-rank test was used to compare the survival distributions. The types of tests are mentioned in each table footnotes. All the *p*-values < 0.05 were considered statistically significant. The patient’s compliance was codified using a binary system as follows: 0 = ICs, 1 = RCs.

For the statistical analysis, the Stata/IC16 (StataCorp. 2019. Stata Statistical Software: Release 16. College Station, TX, USA: StataCorp LLC) software was used [54].

## 3. Results

### 3.1. Demographics

The baseline characteristics of the patients and prosthesis are presented in Table 1 and Table 2, and there were not observed statistically significant differences. Among 76 patients, 41 were women (F) and 35 were men (M). Subjects’ ages ranged from 38 to 76, and the mean age was 57.3 ± 7.7y. Regarding the smoking status, 7 (9.21%) were smokers and 69 (90.79%) were non-smokers (Table 1). The analysis of compliance showed that 51.32% of patients were irregular compliers (ICs), while 37 (48.68%) attended regularly the SPT sessions (RCs). Three patients (3.95%) reported hypertension and ischemic heart disease (HTN, IHD), one patient reported an autoimmune disease (1.32%), and three patients reported diabetes mellitus type II (DM type II) (3.95%). 

In the mandible, 20 prosthesis were identified, while, in the maxilla, 56 prosthesis were identified. From 57 FDPs, 17 (29.82%) were mandible prosthesis, and 40 (70.17%) were maxilla prosthesis. From the 19 RDPs, 3 (15.78%) were mandible prosthesis, and 16 (84.21) were maxilla prosthesis. Regarding the classification of the edentations, 14 subjects (18.42%) in the population were identified with class I Kennedy, 4 (5.26%) with class II Kennedy, 57 (75%) with class III Kennedy, and only 1 (1.32%) with class IV Kennedy, respectively. A subgingival prosthetic preparation was performed in 73 (96.05%) of the subjects, while a supragingival prosthetic preparation was performed in three (3.95%) patients (exclusively for FDPs). The mean number of abutment teeth per patient was 7.11 ± 2.16, while the mean number of pontic units per patient was 3 ± 2.21. From the total number of abutment teeth (541), 436 (80.59%) were present in patients rehabilitated with FDPs, while the rest, 105 (19.40%), were in RDP patients. From 154 pontic units, 127 pontic units were counted in FDP patients, and 27 pontic units were counted in RDP patients. 

### 3.2. Survival Outcome

Fisher’s exact test showed that, overall, there is a statistically significant association between the tooth type and the cause of tooth loss (*p* < 0.05). Thus, in our study population, 57 patients experienced no tooth loss, while 3 patients lost an incisor (2 due periodontal causes and 1 because of a fracture). Furthermore, seven patients lost a premolar (six due to periodontal causes and one due to a fracture); molar extraction was performed in nine patients (eight because of periodontal causes, and one diagnosed with combined periodontal-endodontic lesion) (Table 3).

Furthermore, an overall statistically significant association between existing systemic diseases and the reason for tooth loss was observed (Fisher’s exact test, *p* = 0.033). From 69 patients who did not report a systemic disease, 14 lost a tooth due to periodontal causes, and 2 were fractured and had to be extracted. Only 3 patients from the total of 76 reported a cardiac disease, and from them only 2 lost a tooth due to periodontal causes. One patient reported an autoimmune disease. Regarding diabetes, three patients reported this disease (all with type II), but none had extractions performed (Table 3).

Furthermore, an overall marginally statistically significant association was observed between the smoking status and the reason of tooth loss. Among female subjects [41], 5 subjects lost a tooth for periodontal causes, 1 due to a non-treatable periodontal-endodontic combined lesion, and 2 teeth were fractured, while in male subjects [35], 24 subjects had no tooth extraction, and 11 subjects lost a tooth due to periodontal cause (Table 3). In smokers, from the total of 7 smoking patients, tooth extraction was performed in 1 patient, and 6 had no tooth extraction, while in non-smokers, from the total of 69 non-smoker patients, 51 had no tooth extraction performed, while 17 experienced tooth extraction (15 teeth were lost for periodontal reasons, 1 was diagnosed with a combined endo-perio lesion, and 2 presented fractures). No statistically significant association was made between the smoking status and the reason for tooth loss (Table 3).

Regarding technical complications of the prostheses during the follow-up period, in five patients (6.58%) ceramic fractures were identified, out of which two patients were rehabilitated with RPDs and three with FDPs. Loss of retention was identified in seven (9.21%) patients: four patients were rehabilitated with RDPs and three with FDPs. Mobility of the prostheses (due to mobility of the abutment teeth) occurred in five (6.58%) patients; one patient was rehabilitated with RDP, while four were rehabilitated with FDPs. 

A statistically significant association was identified between technical complications and the type of prostheses (Table 4). In 71 (93.42%) patients no biological complications were identified by clinical or radiographical means. However, in two (2.63%) patients wearing RDPs, endodontic complications were observed on radiographs, even though there was no clinical symptomatology. In addition, in three patients (3.95%, one patient with RDP, and two with FDPs) root caries were detected during the clinical examination.

As seen in Table 5, baseline inter-group comparison between RCs and ICs in terms of periodontal parameters showed no statistically significant differences (unpaired Student’s *t*-test, *p* > 0.05). 

A one-way ANOVA test was conducted to examine the effect that compliance had on PPD, CAL, FMBS, FMPS, and RBL variations in each group. A marginally significant effect of compliance was recorded on FMBS (*p* = 0.05). The rest of the parameters did not record any significant variations due to compliance (*p* > 0.05).

For both ICs and RCs, intra-group analysis between baseline and final examination periodontal parameters showed significant differences on all variables (*p* < 0.05) (Table 6).

**Figure 2:**
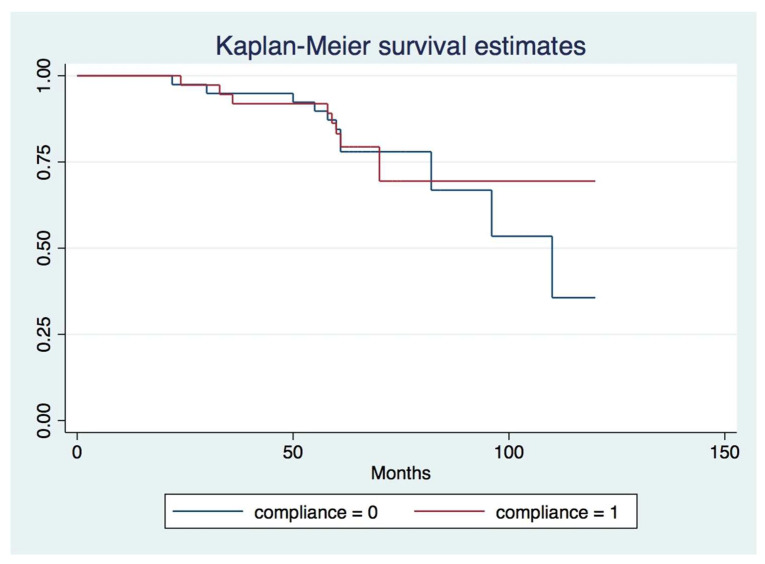
Kaplan–Meier survival curves for ICs (compliance code = 0) and RCs (compliance code = 1).

The Kaplan–Meier survival curves for RCs and ICs are shown in Figure 2. The survival analysis at patient level shows the proportion of subjects with no tooth loss at a given point in time. In our study, the survival curve for the RCs was similar to the ICs for approximately 5 years, during the entire observation period. The log-rank test showed there were no statistically significant differences between survival times between the ICs and RCs (chi-square statistics-0.01, degree of freedom = 1 and *p* value = 0.93). 

## 4. Discussion

The aim of the study was to assess the most relevant factors which are directly involved (alone or in combination) in the long-term survival and functionality of the abutment teeth for extensive stabilizing bridges and removable dental prostheses for treated stage IV periodontitis patients, over a period of 5 year adherence to SPT, with a focus on patient- and prosthesis-related factors. Furthermore, outcomes as survival rates and incidence of biological and technical complications of FDPs and RDPs on abutment teeth with severely reduced, but healthy periodontal tissue support were assessed. In contrast to other authors who focused on investigating the function of prosthetic restorations on dental abutments with advanced bone loss [14,19,25,26,55] or the periodontal conditions at abutment teeth-level [12], our study assessed both the function of the prosthetic restoration and the stability of the outcome of perio-prosthetic therapy in patients treated for Stage IV periodontitis. The main finding of the present study was that provision of extensive stabilizing fixed or removable bridges on teeth with severely reduced periodontal support in patients with Stage IV periodontitis and enrolled in SPT is a successful treatment modality, allowing them to maintain remarkable masticatory abilities over a long period of time. The results of the present study are in line with the findings of previous studies, where advanced loss of the periodontal support around abutment teeth and differences in the design of the FDPs did not influence the periodontal status during the observation period. After follow-ups of 8 to 10 years, 92.17% and 98% of prostheses, respectively, remained in function [19,26]. Despite the severe loss of periodontal tissue support and increased abutment tooth mobility, the teeth observed in the present study could be used successfully as abutments, as 93.43% of the total number of the prostheses they were incorporated into survived for a period of five years, detailed as follows: 89.48% in RDPs and 94.74% in FDPs. 

The comprehensive periodontal treatment, more recently defined as steps 1–3 and step R (prosthetic) by the EFP CPG for periodontitis stages I-III [42] and stage IV [39], respectively, was provided in a private practice in Timisoara, Romania. 

All prostheses were provided by three clinicians with advanced training in prosthodontics and benefited from recent advances, such as preparations under magnification, advanced impression techniques and materials, advanced cementation materials, the replacement of cast frames with laser-sintering-frames, etc. This may explain the reduced failure rate of 6.57% over 5 years (2.63% in RDPs patients and 3.94% in FDPs patients) compared to the overall failure rate of 26% over 14 years in a study reporting about FDPs provided by general practitioners [56].

Particular attention was paid to the width of the interproximal areas in order to prevent them from being over-contoured, in order to favor optimal interdental hygiene and the teeth which presented furcation [57]. Even though some studies have shown that a supragingival location of the crown margin is more favorable in preventing gingival inflammation, when compared with a subgingival location [53,58], several studies showed that partial crowns in comparison to full crowns have reduced resistance to deformation. The ultimate consequence of plastic deformation of a crown in a bridge is the fracture of the luting cement and, therefore, loss of retention [26]. In our study population, only 5.27% (3/76) of the prosthetic preparations were performed supragingivally, all in FDPs patients, and none of them were lost. Furthermore, the rigid splint of the abutment teeth through extensive FDPs with a correct occlusal design reduced the risk of increasing mobility. Furthermore, one of our concerns was to avoid the progressive mobility of the bridgework with the time, or to preserve the status quo *of bridge stability*, as formulated by Nyman and Lindhe in 1979 [26]. Another elementary principle in attaining a long-time survival of these bridges was to obtain a proper retention, meaning that it was necessary to provide the prepared portion of an abutment tooth with maximal length and minimal taper [59]. Furthermore, in our subjects, FDPs with cantilevers were completely avoided, because, as numerous studies have shown, the danger of abutment teeth fracture in this type of constructions was twice as frequent at abutments adjacent to cantilevers, when compared to abutments not adjacent to cantilevers [55,60,61,62]. 

Due to the severity of the periodontal support tissue loss which, in many cases, led to elongation of the clinical crown and pathological tooth migrations, and because of the prevalence of subgingival prosthetic preparation in the patient population, most of the abutment teeth had root canal treatment performed. Despite controversies in the literature regarding the opportunity of pre-emptive root canal treatments in abutment teeth [63,64,65], teeth in patients with Stage IV periodontitis, such as in our study population, frequently necessitate actual prosthodontic preparation of the root itself, which interferes with the pulpal space. This explains the relatively high supragingival preparations where the aesthetic situation allowed. Teeth showing incorrect root canal treatment or periapical lesions at radiographic examination were endodontically re-treated before the prosthetic treatment. This may be the reason of the low incidence rate of endodontic complications in our study (2.63%, all of them in RDPs patients), compared to a study undertaken in a population with advanced periodontal disease, where the incidence of endodontic complication was 15% [65]. 

Attention should be paid when comparing the results obtained for RDPs in our study with the results of previous studies. In our particular case, RDPs referred to hybrid constructions with a fixed, cemented component and a removable component anchored to the fixed component by precision attachments, while overdentures were completely excluded.

Compared to another long-term study on tooth loss in periodontally compromised patients [20] in which 76% of the patients were fully compliant with SPT intervals, in our population, only 48.68% of the patients were fully compliant with the recommended 3-month SPT intervals. Nevertheless, our results are comparable with another study from 2008, where the authors reported a rate of compliance of 54% [1]. 

When taking the discussion at the tooth level, of the total abutment teeth, only 3.51% (19/541) were lost at the end of the observation period in our study, and the causes were identified as follows: 84.21% (16/19) were lost because of progression of periodontal disease, a root fracture was identified in 10.52% (2/19) of the extracted abutment teeth, and 5.26% (1/19) were diagnosed with a combined endodontic-periodontal lesion. For patients rehabilitated with RDPs, another study from 2015 reported that 15% of the total abutment teeth were lost at the end of the observation period, 42% were lost because of periodontal disease, 29.6% were extracted to manage dental caries, and 26.5% were identified as tooth fractures [66], while in our study only 3.73% of the total number of abutment teeth in RDP patients were lost, for 25% a root fracture was identified, and 75% were lost for periodontal cause. Additionally, a previous report showed that approximately 18.8% of teeth used as abutments for RPDs in periodontal patients were extracted because of tooth fracture [67]. While our results regarding teeth fractures seem to be in line with the aforementioned, representative studies, the differences regarding the teeth lost for periodontal reasons may be attributed to the type of RPD used (dentures with telescopic retainers vs. extra-coronal precision attachments in our study), and to the severity of the disease (Stage IV) in patients included in our study, respectively. 

Patients diagnosed with Stage IV periodontitis and rehabilitated with FDPs or RDPs who were evaluated in our study lost a total of 19/541 (3.51%) teeth during a mean observation period of 69.01 months (5.75 years) after prosthetic rehabilitation. That is a mean loss of 0.25 teeth per patient or 0.04 teeth per patient per year. These results are comparable with those reported by another study in patients with RDPs and FDPs, which showed a mean tooth loss of 3.5 teeth per patient and 0.36 tooth loss per patient and year [20]. However, the rate of tooth loss observed in our population is lower than the rate reported in other studies, where the rate of tooth (abutment) loss in FDPs was 12%, and further increased to 18% in RPDs [1]. This may be due to the longer period of follow-up (10 years vs. 5 years in our study), and to the reduced extent of the FDPs (3–5 units in the mentioned study vs. 6+ in our study, which obviously provides a better polygon of support). 

Technical complications occurred in 17 patients during the observation period. In five cases, ceramic fracture was observed, which was judged minor by the patients and did not cause concern. In seven cases, the prosthetic reconstruction became loose and had to be removed and recemented. In the remaining cases, mobility of the reconstruction could be observed. One reason for the increased complication rate in the present study could be the unfavorable abutment-to-pontic ratio, which was lower than the one reported in a 10-year retrospective study where, in 94 bridgeworks only 7 technical complications were identified (2 cases with ceramic fracture, 4 bridges became loose and were recemented and 1 framework fracture) [19].

However, an overall statistically significant association was identified between biological complications and the type of prostheses. Although, in 71 out of 76 patients no biological complications could be identified by clinical or radiographical means, in two (2.63%) patients, endodontic complications were observed on radiographs, without clinical symptomatology. In addition, in three patients, root caries were detected during the clinical examination. Our results are comparable to the results of the aforementioned study, where the authors reported that eight abutment teeth were lost, seven due to caries, and one due to endodontic complications [19].

In five (6.57%) cases, abutment tooth loss resulted in the loss of the prosthodontic reconstruction; two (2.63%) cases were in patients with RDPs, and three (3.94%) were in FDP patients. This is comparable with the results of another study from 2013, where the authors reported that in patients with FDPs and RDPs, abutment extraction led to the loss of 3.55% of the prosthodontic reconstructions at the end of the observation period [20].

Our findings show that, although the survival rates at patient level remained above 80% for approximately 5 years, the survival curves for the RCs reported a surprisingly similar outcome to the ICs group. This is in line with the results of a recent study, where patients with RDPs and attending SPT at an interval of 3–6 months (in other words, RCs) showed similar survival curves with patients who attended SPT at 1-year intervals [66]. Thus, it seems pertinent to discuss the fact that our analysis failed to identify irregular attendance to SPT as a risk factor for tooth loss. We are assuming that definition of ICs plays a decisive role when the effect of SPT is to be analyzed, because our study did not include patients who discontinued therapy, whereas other studies did [1,32], and the recall intervals seem to be more important than the regularity of the SPT sessions. 

The results of this study show statistically significant correlations between the presence of cardiovascular diseases (in our study, mainly hypertension) and tooth loss, and between the type of tooth lost and tooth loss, irrespective of the type of prosthesis. This can be explained by the effects induced by the anti-hypertensive medication [68], and by the relatively increased vulnerability of multirooted teeth facing the progression of attachment loss, respectively. Furthermore, the gender of the subjects was marginally statistically significant associated with the tooth loss, however, no explanation could be given to this finding. Interestingly, in our population, smoking is not associated with tooth loss; thus, it can be speculated that a regular SPT in most of the patients probably reduced the negative effect of this habit. In fact, this finding was reported in another study, as well [21]. 

The present study has several limitations. The retrospective analysis of data has a limited level of evidence, since the bias of selection and the reporting bias could not be completely excluded. We tried to overcome this limitation by selecting consecutive patients, and, thus, reducing the bias. Furthermore, the patients in this study were limited to those attending a university periodontal clinic, and, therefore, this might be a more selective sample. A limitation of the study might also be considered in terms of the medium-term follow up (a mean follow-up period of 69.01 ± 15.39 months), making future studies on a longer period of time necessary.

## 5. Conclusions

If a comprehensive and systematic periodontal treatment is performed prior to the prosthetic rehabilitation, and regular consequent SPT is implemented, a successful medium-term retention of periodontally compromised teeth is possible for patients diagnosed with Stage IV periodontitis, through extended prosthetic restoration including these teeth, with predictable long-term results, regardless of the type of stabilizing prosthesis. After five years, 93% of the prosthesis were still in place and functional, with reduced rates of technical and biological complications. 

## Figures and Tables

**Table 1:** Patients’ characteristics and demographics.

	Frequency	Percent
Sex (n, %)		
F	41	53.95
M	35	46.05
Age (years, mean ± SD)		
	57.3 ± 7.70	
Smoking Status (N, %)		
Smoker	7	9.21
Non-smoker	69	90.79
Compliance		
ICs	39	51.32
RCs	37	48.68
Systemic Disorders (N, %)		
Unreported	69	90.79
Cardiovascular (HTN, IHD)	3	3.95
Autoimmune	1	1.32
DM type II	3	3.95

Abbreviations are as follows: RCs (regular compliers)—patients attending 100% of SPT visits; ICs (irregular compliers)—patients that missed scheduled regular visits, but continued irregularly; HTN—hypertension; IHD—ischemic heart diseases; DM type II—diabetes mellitus type II.

**Table 2:** Characteristics of the prosthesis.

	RDPs (19)	FDPs (57)
Compliance N(%)		
ICs	11 (57.89)	28 (49.12)
RCs	8 (42.10)	29 (50.87)
Jaw, N(%)		
Mandible	3 (15.78)	17 (29.82%)
Maxilla	16 (84.21)	40 (70,17%)
Type of preparation, N(%)		
Supragingial	-	3 (5.26)
Subgingival	19 (100)	56 (94.74)
Edentation according to Kennedy’s Classification, N(%)		
Class I	14 (73.6)	-
Class II	4 (21.05)	-
Class III	1 (0.52)	56 (94.73)
Class IV	-	1 (1.75)
Usage period (months, mean± SD)	71.9371.93 ± 17.88	66.84 ± 14.15
Abutment teeth, N	105	436
Pontic units, N	27	127

Abbreviations are as follows: RDPs—removable dental prostheses; FDPs—fixed dental prostheses; RCs—regular compliers; ICs—irregular compliers.

**Table 3:** Characteristics of teeth and population related to causes underlying tooth loss during SPT.

	No Tooth Loss (N, %)	Periodontal (N, %)	Endo-Perio (N, %)	Fracture (N, %)	Overall *p*-Value
Type of tooth					
No tooth loss	57 (100)	-	-	-	* 0.000
Incisor	-	2 (66.67)	0 (0.00)	1 (33.33)	
Premolar	-	6 (85.71)		1 (14.29)	
Molar	-	8 (88.89)	1 (11.11)	-	
Smoking status					
Smoker	6 (85.71)	1 (14.29)	-	-	1.000
Non-smoker	51 (73.91)	15 (21.74)	1 (1.45)	2 (2.90)	
Systemic diseases					
Reported as healthy	53 (76.81)	14 (20.29)	-	2 (2.90)	* 0.033
Cardio-vascular	1 (33.33)	2 (66.67)	-	-	
Autoimmune			1 (100)		
DM type II	3 (100)	-	-	-	
Sex					
Female	33 (80.49)	5 (12.20)	1 (2.44)	2 (4.88)	0.067
Male	24 (68.57)	11 (31.43)	-	-	

Fisher’s exact test; * Statistically significant.

**Table 4:** Description of technical and biological complications by the type of prostheses.

	RDPs N, (%)	FDPs N, (%)	*p*-Value
Technical complications			
Ceramic fracture	3 (10.53)	3 (5.26)	0.108
Mobility	4 (5.26)	4 (7.02)	
Loss of retention	3 (21.05)	3 (5.26)	
No complications	47 (63.16)	47 (82.46)	
Biological complications			
No complications	16 (84.21)	55 (96.49)	0.04 *
Root caries	1 (5.26)	2 (3.51)	
Endo Complications	2 (10.53)	-	

Fisher’s exact test; * Statistically significant. Abbreviations are as follows: FDPS—fixed dental prostheses; RDPS—removable dental prostheses; *P*-value—overall *p*-value.

**Table 5:** Baseline and final examination intergroup comparisons between RCs and ICs regarding periodontal parameters.

		PPD	CAL	FMBS	FMPS	RBL
Baseline	ICs	2.85 ± 0.30	3.10 ± 0.46	16.92 ± 5.09	9.94 ± 5.37	60.1 ± 9.58
	RCs	2.76 ± 0.29	3.28 ± 0.55	15.08 ± 7.20	9.54 ± 5.28	63.10 ± 9.14
*p*-value ^a^		0.16	0.12	0.20	0.73	0.17
Final examination	ICs	3.56 ± 0.53	3.90 ± 0.66	31.07 ± 13.36	16.33 ± 7.56	65.97 ± 14.87
	RCs	3.31 ± 0.53	3.92 ± 0.62	24.40 ± 11.76	13.70 ± 8.53	68.13 ± 9.87
*p*-value ^a^		0.04 *	0.88	0.02 *	0.15	0.46
Difference to baseline	ICs	0.70 ± 0.40	0.8 ± 0.52	14.15 ± 11.50	6.38 ± 4.39	5.84 ± 9.63
	RCs	0.55 ± 0.38	0.64 ± 0.38	9.32 ± 10.02	4.16 ± 6.57	5.02 ± 3.73
*p*-value ^b^		0.63	0.10	0.05	0.13	0.08

* Statistically significant. ^a^ Unpaired t-test; for inter-group comparison. ^b^ One-way ANOVA for intra-group variations. PPD, CAL, FMBS, and FMPP represent the overall mean values per patient for the entire group.

**Table 6:** Baseline and final examination intra-group comparisons among RCs and ICs regarding periodontal parameters.

	ICs		*p*-Value	RCs		*p*-Value
	T0	T1		T0	T1	
PPD	2.85 ± 0.30	3.56 ± 0.53	0.00 *	2.76 ± 0.29	3.31 ± 0.53	0.00 *
difference	0.70 ± 0.40			0.55 ± 0.38		
CAL	3.10 ± 0.46	3.90 ± 0.66	0.00 *	3.28 ± 0.55	3.92 ± 0.62	0.00 *
difference	0.8 ± 0.52			0.64 ± 0.38		
FMBS	16.92 ± 5.09	31.07 ± 13.36	0.00 *	15.08 ± 7.20	24.40 ± 11.76	0.00 *
difference	14.15 ± 11.50			9.32 ± 10.02		
FMPS	9.94 ± 5.37	16.33 ± 7.56	0.00 *	9.54 ± 5.28	13.70 ± 8.53	0.00 *
difference	6.38 ± 4.39			4.16 ± 6.57		
RBL	60.12 ± 9.58	65.97 ± 14.87	0.00 *	63.10 ± 9.14	68.13 ± 9.87	0.00 *
difference	5.84 ± 9.63			5.02 ± 3.73		

Paired Student t test; * Statistically significant. Here, PPD, CAL, FMBS, FMPS present overall mean values per patient for the entire group.

## Data Availability

Data are available from the corresponding author upon reasonable request.
